# Supplementary material for: Redox-informed models of global biogeochemical cycles
Source: Nat Commun. 2020 Nov 10;11:5680. doi: 10.1038/s41467-020-19454-w (PMC7656242; doi:10.1038/s41467-020-19454-w)
Supplement: Supplementary file 1 — Supplementary Information [file 41467_2020_19454_MOESM1_ESM.pdf]

# Supplementary Information

Redox-informed models of global biogeochemical cycles

Zakem et al.

# Contents: Supplementary Notes 1-4, Supplementary Figures 1-4

## Supplementary Note 1:

The redox-informed methodology is useful for quantifying the ballpark differences between distinct metabolisms (i.e. comparing aerobic heterotrophy to chemoautotrophic ammonia oxidation as in Table 1 in the main text). Due to variation in cellular allocation towards the machinery for uptake vs. growth, among other demands, other constraints must be added to understand the differences among populations carrying out any one of these metabolisms. For example, there exists a trade-off between uptake rate and yield due to a finite proteome. An example of this is the well-known trade-off between growth rate and yield of ATP production when considering aerobic respiration vs fermentation of a substrate[1–3]. Aerobic respiration maintains a higher ATP yield, but must allocate enzyme towards the cellular machinery required to use oxygen, while fermentation sacrifices the higher yield and allocates enzyme towards the machinery for faster substrate uptake[2].

Integration with trait-based modeling approaches can incorporate this trade-off into the framework. For example, the yield $y$ (moles biomass synthesized per mole substrate utilized) and specific uptake rate $V$ ($t^{-1}$) in Eqn. 2 (in Box 1 in the main text) determine the population's fitness in an environment, and thus we may consider them as traits. For example, aerobic respiration can be described with a lower maximum uptake rate and a higher yield as compared to fermentation, with the finite proteome used to quantitatively link the two traits.

This tradeoff between uptake rate and yield corresponds to the dichotomy of "opportunists" vs. "gleaners," respectively[4], or, "r-selected" vs. "K-selected" species from the logistic equation for population dynamics[5]. When using a Michaelis-Menten form to describe uptake of resource $R$ with maximum specific uptake rate $V_{max}$ and half-saturation concentration $k_S$, the resource subsistence concentration ($R^*$)[4,6,7] is

$$R^* = \frac{k_S L}{y V_{max} - L} \qquad \text{(S1)}$$

where $yV_{max}$ is the maximum growth rate and $L$ is the population loss rate. Supplementary Fig. 1 illustrates the trade-off with two functional types – an "opportunist" and a "gleaner" – competing for substrate in a virtual chemostat. Though the yield $y$ is the same, the opportunist has a higher maximum growth rate and a lower substrate affinity than the gleaner, which has a lower $R^*$. When substrate is supplied intermittently, one or the other may be excluded over time, or both may be sustained if the variable conditions prevent competitive exclusion entirely (as in Supplementary Fig. 1). In reality, we can understand that these tradeoffs may represent characteristics among populations carrying out a similar metabolism, and that conditions may select temporarily the most optimized from the species pool so that dynamic change can be expected on short time scales.

**Supplementary Note 2:**

The redox-informed biomass yield is useful even if models of substrate uptake are empirical. For a heterotroph, for example, the yield determines the amount of substrate that is transformed and excreted as a respiration product such as $CO_2$. In our example of the nitrification ecosystem, the redox-informed differences in the yield between ammonia and nitrite oxidizers have provided useful explanations for the observed differences between ammonia and nitrite concentrations and the biomasses of the two clades, despite uncertainty in uptake kinetics.

We can demonstrate the utility of the yield alone using a simple system for substrate concentration $S$ and consuming biomass $B$:

$$\frac{dS}{dt} = S_{\text{in}} - V(S)B \quad \text{(S2)}$$

$$\frac{dB}{dt} = yV(S)B - LB \quad \text{(S3)}$$

where $S_{\text{in}}$ is the substrate supply rate, $V(S)$ is the specific uptake rate function that depends on the substrate concentration, $y$ is the yield, and $L$ is the loss rate. At steady state, we can solve for the steady concentration of biomass $B^*$ as:

$$yS_{\text{in}} = LB^* \quad \text{(S4)}$$

$$B^* = y^{-1}LS_{\text{in}} \quad \text{(S5)}$$

The steady state biomass is proportional to the inverse of the yield, and the uptake rate falls out of the equation. Therefore, the model can be useful in relating quantities of biomass to substrate supply, independent of uptake kinetics. Differences in yield alone also result in differences in the subsistence concentrations (Eqn. S1).

**Supplementary Note 3**:

Recent data suggests that nitrite-oxidizing bacteria are more efficient with their allotted energy supply than ammonia-oxidizing archaea[8]. However, the data still agree with the redox-informed biomass yields in terms of assimilation per amount of N oxidized, and thus that differences in yields and cell size can explain much of the difference in abundance.

To explain, Kitzinger et al. 2020 calculate per cell oxidation rates for ammonia-oxidizing archaea (AOA) and nitrite-oxidizing Nitrospinae bacteria (NOB) in the Gulf of Mexico[8]. They find that the per cell nitrite-oxidation rates were ~15-fold higher than the per cell ammonia-oxidation rates. Per cell N assimilation rates were 0.91 for NOB and 0.12 for AOA, thus 7.5-fold higher for the larger NOB. Comparing the per cell nitrification rates to the per cell N assimilation rates provides the best test of the redox-based theoretical model, which predicts a three-fold difference in biomass yield in terms of biomass assimilation per mol N oxidized[9]. Together, these observations suggest that the biomass yield of AOA is approximately two-fold higher than the biomass yield of NOB (mol N assimilated per mol N oxidized), and so observations are 33% lower than the theoretical three-fold prediction.

Kitzinger et al. evaluate an efficiency in different terms: moles of carbon fixed per Joule (and converting from N assimilation with a constant elemental ratio). Our theoretical model predicts that the efficiency in these terms (moles of carbon fixed per Joule) is relatively similar for the two nitrifying populations. Specifically, the model relates the bulk ammonia oxidation rate ($R_{AO}$; nmol $NH_4^+$ $L^{-1}$ $d^{-1}$) and bulk nitrite oxidation rate ($R_{NO}$; nmol $NO_2^-$ $L^{-1}$ $d^{-1}$) to cellular rates as:

$$R_{AO} = y_{AOA}^{-1} \mu_{AOA} B_{AOA} \quad \text{(S6)}$$
$$R_{NO} = y_{NOB}^{-1} \mu_{NOB} B_{NOB} \quad \text{(S7)}$$

for yield $y$ (mol biomass per mol N oxidized), growth rate $\mu$ ($d^{-1}$), and biomass $B$ (mol C or N $L^{-1}$). This shows that deviations from the steady state where $R_{AO} = R_{NO}$ can result from changes in the relative growth rates or biomasses between the two populations if yields remain relatively stable over time.

Kitzinger et al. calculate the efficiency in terms of moles of carbon fixed per Joule by multiplying the above per cell nitrification rates by the Gibbs free energies of reactions as:

$$E_{AOA} = \Delta G_{AO} R_{AO} A_{AOA}^{-1} \quad \text{(S8)}$$
$$E_{NOB} = \Delta G_{NO} R_{NO} A_{NOB}^{-1} \quad \text{(S9)}$$

for assimilation $A$ (mol C assimilated $L^{-1}$ $d^{-1}$) and free energy release $\Delta G$ (kJ per mol N oxidized). In the model framework, $A = \mu B$, and therefore:

$$E_{AOA} = \Delta G_{AO} y_{AOA}^{-1} \quad \text{(S10)}$$
$$E_{NOB} = \Delta G_{NO} y_{NOB}^{-1} \quad \text{(S11)}$$

Thus, if the difference in yield is relatively equal to the difference in free energies, as is predicted theoretically, this difference cancels out in Eqns. S10 and S11, and the efficiencies in terms of assimilation per Joule are predicted to be relatively equal for the two populations. However, in their calculation, Kitzinger et al. calculate efficiencies using Eqns. S8 and S9 using measurements from a dynamic environment where $R_{AO}$ is four-fold higher than $R_{NO}$ (a large departure from a steady state), and they calculate a four-fold higher efficiency. Their calculation also assumes that the 7.5-fold higher assimilation rate of NOB applies *in situ*.

**Supplementary Note 4:**

The metabolic functional type approach can mechanistically represent microbial activity in dynamic steady-state or time-varying environments. This predictive power is of particular benefit for resolution of microbial processes in fine-grained ocean circulation models where flow can vary on the order of days, similar to the timescales of microbial growth. However, solutions become dependent on the partitioning of metabolism among the functional types as the timescales of physical change approach the timescales of microbial growth.

For example, Supplementary Fig. 3 illustrates a model simulating a zonal transect through the S. Pacific Ocean, which contains an anoxic oxygen minimum zone. The ecosystem model resolves emergent metabolic activity along a gradient in oxygen with (for simplicity of illustration) just two resolved microbial metabolisms: aerobic and anaerobic (denitrifying: $NO_3^- \rightarrow N_2$) heterotrophy. The equilibrium state solutions of two versions of this model – one with two obligate (aerobic and anaerobic) populations and the other with one facultatively anaerobic population – are nearly identical at steady state, but different in the transient.

Supplementary Fig. 4 illustrates the differences in the time evolution of the oxygen concentration and the denitrification rate following a perturbation in which oxygen concentrations are increased. In the model with the two obligate types, anaerobic denitrification does not cease throughout the recovery period because a population of anaerobes remains after $O_2$ is supplied and competitive exclusion has not come to completion. In contrast, the facultative population switches to respiring $O_2$ when it is supplied, and so denitrification rates cease immediately. Real community dynamics likely reflect both solutions: current understanding is that heterotrophic microorganisms are generally facultatively anaerobic, while chemoautotrophic anaerobic ammonium oxidizing (anammox) bacteria, which accounts for roughly a third of the fixed N loss in pelagic zones, are understood to be obligate anaerobes[10–13].

Thus, knowledge of how metabolisms are distributed among populations is required for interpreting transient states. Other species-specific time-varying phenomena such as the lag response of organisms to substrate availability also becomes relevant[14]. These are limitations on one hand, and so require further constraint. On the other hand, these differences may serve as a tool for parsing out how metabolisms are distributed by comparing models with observed time series. The different solution states in Supplementary Fig. 4, for example, can serve as testable hypotheses against which the response of natural assemblages can be compared.

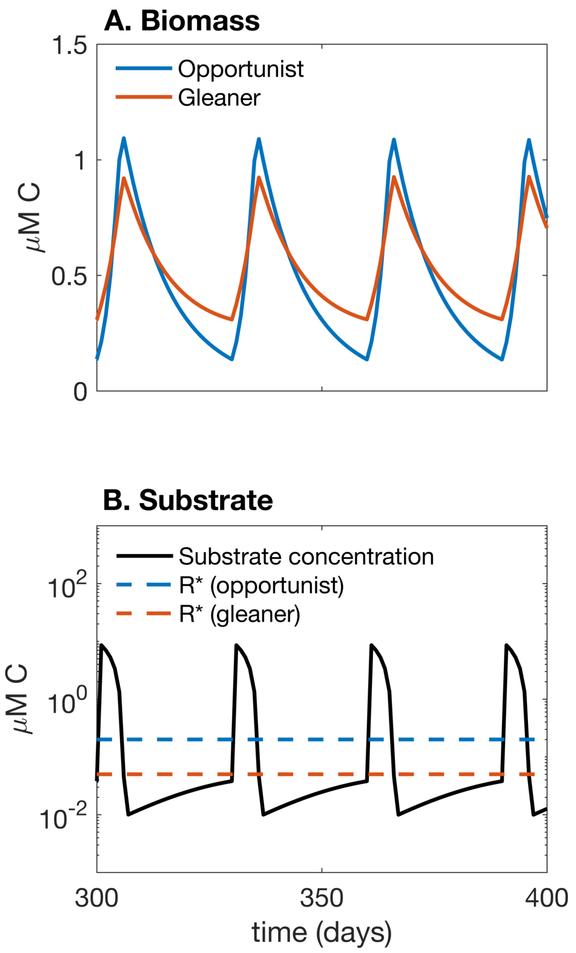


**Supplementary Figure 1:** A model simulation of the competition between two functional types characterized by a tradeoff among traits, with **A**. resulting biomasses and **B.** substrate concentration. The "opportunist" has a higher maximum growth rate and a lower substrate affinity than the "gleaner," which has a lower subsistence concentration ($R^*$). The two populations grow in a virtual chemostat with dilution rate 0.1 $d^{-1}$, with a continual incoming substrate concentration of 1 µM C and an additional 10 µM C pulse of substrate added every 30 days. Here, the "opportunist" is parameterized with a maximum uptake rate of $V_{max}$ = 2 mol substrate C per mol biomass C per day and a half-saturation concentration $k_S$ = 1 µM C. For the "gleaner," has $V_{max}$ = 1 mol substrate C (mol biomass C)$^{-1}$ $d^{-1}$ and $k_S$ = 0.1 µM C. For both, substrate yield $y$ = 0.3 mol biomass C (mol substrate C)$^{-1}$.

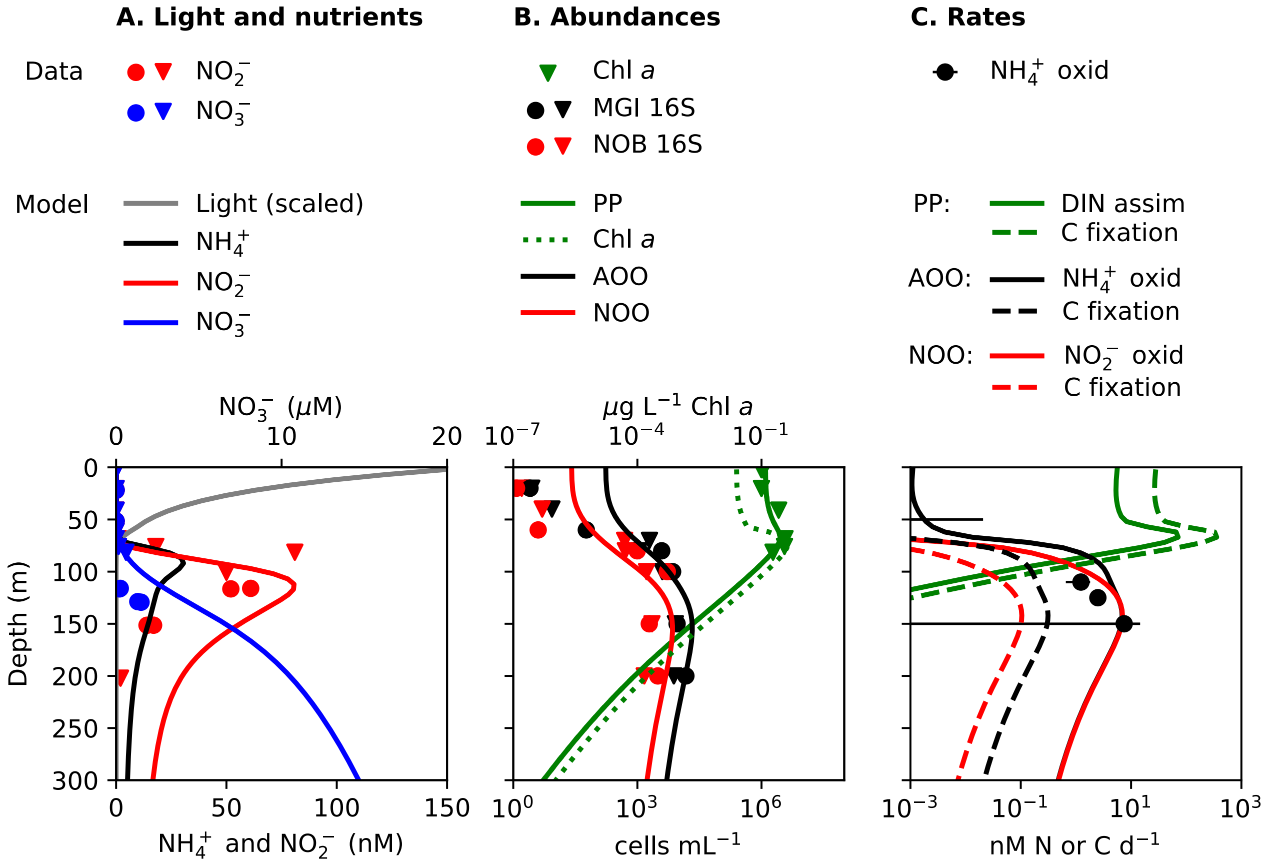


**Supplementary Figure 2:** Model simulation of the nitrification system in the North Pacific. Lines are model solutions[9], and marked points are observations from Santoro et al. 2010 and Santoro et al. 2013 (cruise stations 67.115 (triangles) and 67.155 (circles) in the Pacific Ocean[15,16]). **A.** Light (normalized to the width of the box) and dissolved inorganic nitrogen species. **B.** Cell abundances, converting from modeled biomass concentration assuming 0.1 fmol N cell$^{-1}$ and one gene copy per cell for phytoplankton (PP), ammonia-oxidizing organisms (AOO), and nitrite-oxidizing organisms (NOO). Note that the conversion differs here from Fig. 4 in the main text. Here (unlike in the main text), we show results with the same cell quota (0.1 fmol N cell$^{-1}$) for all populations, which also have the same cell-size-based uptake kinetic parameters, in order to isolate the impact of the underlying redox chemistry on the solutions. Observed abundances are of the 16S rRNA abundances of archaeal Marine Group I (MGI) and of *Nitrospina*-like organisms (NOB). **C.** Rates of N cycling and C fixation by the three autotrophic functional types. The equal N-cycling rates are consistent with observations that show indistinguishable rates of ammonia and nitrite oxidation below the euphotic zone in steady environments[17].

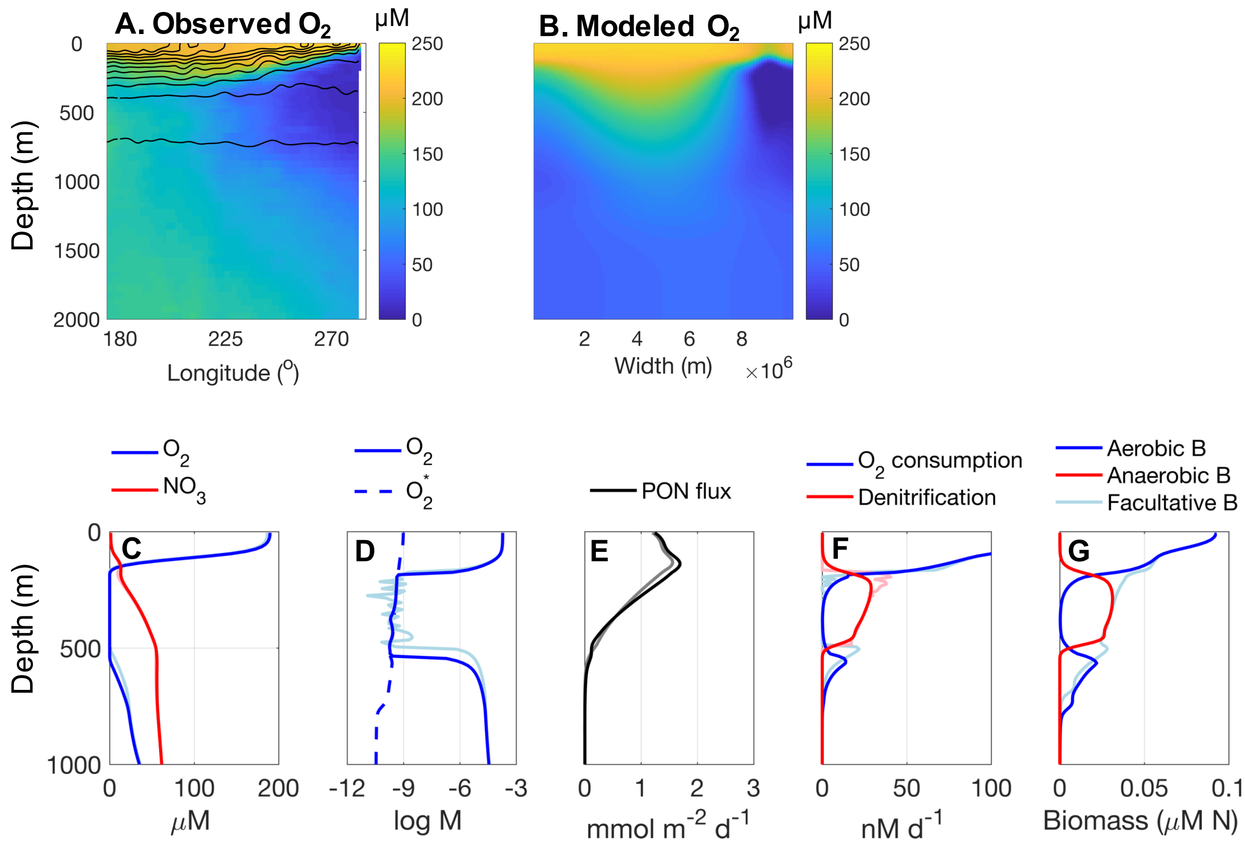


**Supplementary Figure 3: Ecosystem model of the 10°S transect across the South Pacific Ocean.** Heterotrophic microbial functional types consume and respire organic matter with $O_2$ or DIN (dissolved inorganic nitrogen) as an electron acceptor, with no prescribed critical or inhibiting oxygen concentrations. **A.** [$O_2$] from the World Ocean Atlas (2013) with lines depicting isopycnal (density) surfaces[18]. **B.** Modeled steady state [$O_2$]. **C-G**. A profile of the solutions through the anoxic zone. Darker lines show solutions for a model version with two distinct metabolic functional type populations: obligate aerobic and anaerobic (denitrifying) heterotrophs. Lighter lines show solutions for a second model version with one facultatively aerobic population that consumes either [$O_2$] or DIN, depending on which allows for a higher growth rate at each time step in the model. For the respiration rates of the facultative type (**F**), averages over 100 days at steady state are illustrated.

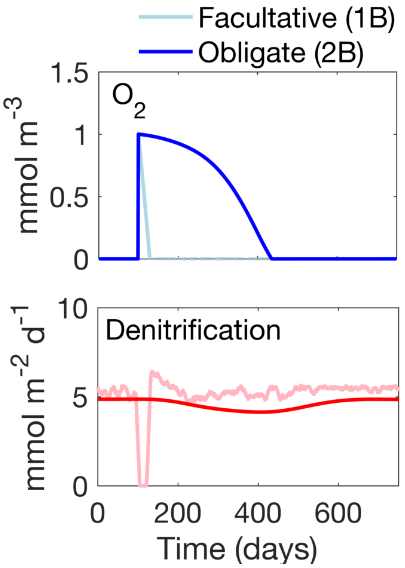


**Supplementary Figure 4.** The time progression of [$O_2$] in the core of the simulated anoxic zone (Supplementary Fig. 2) and the integrated denitrification rate, with a perturbation at 100 days in which the model [$O_2$] minimum was raised to 1 μM.

## Supplementary References


1. Pfeiffer, T., Schuster, S. & Bonhoeffer, S. Cooperation and competition in the evolution of ATP-producing pathways. *Science* **292**, 504–7 (2001).
2. Basan, M. *et al.* Overflow metabolism in Escherichia coli results from efficient proteome allocation. *Nature* **528**, 99–104 (2015).
3. Roller, B. R. K., Stoddard, S. F. & Schmidt, T. M. Exploiting rRNA operon copy number to investigate bacterial reproductive strategies. *Nat. Microbiol.* **1**, 1–7 (2016).
4. Grover, J. P. Resource competition in a variable environment: Phytoplankton growing according to the variable-internal-stores model. *Am. Nat.* **138**, 811–835 (1991).
5. MacArthur, R. H. & Wilson, E. O. *The theory of island biogeography*. (Princeton University Press, 1967).
6. Stewart, F. M. & Levin, B. R. Partitioning of resources and the outcome of interspecific competition: A model and some general considerations. *Am. Nat.* **107**, 171–198 (1973).
7. Tilman, D. *Resource competition and community structure*. (Princeton University Press, 1982).
8. Kitzinger, K. *et al.* Single cell analyses reveal contrasting life strategies of the two main nitrifiers in the ocean. *Nat. Commun.* **11**, 767 (2020).
9. Zakem, E. J. *et al.* Ecological control of nitrite in the upper ocean. *Nat. Commun.* **9**, 1206 (2018).
10. Kartal, B., Keltjens, J. T. & Jetten, M. S. M. The metabolism of anammox. in *Encyclopedia of Life Sciences (ELS)* (John Wiley and Sons, 2008).
11. Ward, B. B. Oceans. How nitrogen is lost. *Science* **341**, 352–3 (2013).
12. Zumft, W. G. Cell biology and molecular basis of denitrification. *Microbiol. Mol. Biol. Rev.* **61**, 533–616 (1997).
13. Koeve, W. & Kähler, P. Heterotrophic denitrification vs. autotrophic anammox - quantifying collateral effects on the oceanic carbon cycle. *Biogeosciences* **7**, 2327–2337 (2010).
14. Klappenbach, J. A., Dunbar, J. M. & Schmidt, T. M. rRNA operon copy number reflects ecological strategies of bacteria. *Appl. Environ. Microbiol.* **66**, 1328–1333 (2000).
15. Santoro, A. E., Casciotti, K. L. & Francis, C. A. Activity, abundance and diversity of nitrifying archaea and bacteria in the central California Current. *Environ. Microbiol.* **12**, 1989–2006 (2010).
16. Santoro, A. E. *et al.* Measurements of nitrite production in and around the primary nitrite maximum in the central California Current. *Biogeosciences* **10**, 7395–7410 (2013).
17. Ward, B. B. Nitrification in marine systems. in *Nitrogen in the Marine Environment* (eds. Capone, D. G., Bronk, D. A., Mulholland, M. R. & Carpenter, E. J.) 199–262 (Academic Press, 2008).
18. Garcia, H. E. *et al.* World Ocean Atlas 2013. Vol. 3: Dissolved oxygen, apparent oxygen utilization, and oxygen saturation. *Tech. Ed. NOAA Atlas NESDIS 75* **3**, 27 pp. (2013).
